# Assessment of Sealing Ability and Degradation Resistance of a Hydrogel‐Based Root Canal Filling Material Using a Bacterial Leakage Model and SEM Analysis

**DOI:** 10.1155/bmri/6718265

**Published:** 2026-06-16

**Authors:** Else Ellermann, Daniel Richter, Ignasi Belda Punzano, Andreas Schmocker, Mark Bispinghoff, Tan Fırat Eyüboğlu, Mutlu Özcan

**Affiliations:** ^1^ Odne AG, Dübendorf, Switzerland; ^2^ Laboratory of Inorganic Chemistry, ETH Zurich, Zurich, Switzerland, ethz.ch; ^3^ Clinic of Masticatory Disorders and Dental Biomaterials, Center for Dental Medicine, University of Zurich, Zurich, Switzerland, uzh.ch; ^4^ Department of Neuroradiology, Inselspital, Bern University Hospital, University of Bern, Bern, Switzerland, unibe.ch; ^5^ Department of Endodontics, Faculty of Dentistry, Istanbul Medipol University, Istanbul, Türkiye, medipol.edu.tr

**Keywords:** bacterial leakage, dental materials, hydrogel-based root canal filling material, microleakage

## Abstract

**Background:**

This study evaluated the sealing ability and degradation resistance of a novel hydrogel‐based, low‐viscosity, light‐curable obturation material compared with gutta‐percha combined with an epoxy resin–based sealer using a bacterial leakage model and scanning electron microscopy (SEM) analysis.

**Methods:**

Thirty‐seven extracted single‐rooted bovine incisors were allocated into four groups: gutta‐percha + epoxy resin sealer (*n* = 12), hydrogel‐based material (*n* = 10), positive control with empty canals (*n* = 11), and negative control (*n* = 4). Bacterial leakage was assessed using a two‐chamber model inoculated with *Enterococcus faecalis*. Survival times were analyzed using nonparametric tests. For degradation analysis, 30 hydrogel specimens (*n* = 6 per medium) were incubated in phosphate‐buffered saline (PBS), brain heart infusion (BHI) broth, *E. faecalis* suspension, sterile‐filtered saliva, or pH 10 carbonate buffer. SEM‐based Feret diameter measurements were obtained after 1, 8, and 30 days and analyzed using Kruskal–Wallis and Holm‐adjusted Mann–Whitney *U* tests.

**Results:**

The first leakage occurred after 11 days in the gutta‐percha group and after 58 days in the hydrogel group. Forty percent failure was observed after 22 days for gutta‐percha and after 121 days for the hydrogel material (*p* < 0.001). Mean Feret diameter increased most markedly under alkaline conditions (*Δ* = +0.100 * μ*m from Day 1 to Day 30; *p* < 0.001), whereas changes in PBS, BHI, *E. faecalis*, and saliva remained below 0.05 *μ*m over 30 days.

**Conclusions:**

The hydrogel‐based obturation material demonstrated significantly prolonged resistance to bacterial penetration and maintained structural stability under biologically relevant incubation conditions. Further long‐term and in vivo studies are required to confirm clinical performance.

## 1. Introduction

Root canal treatment is aimed at eliminating infection and preventing reinfection of the root canal system while preserving the natural tooth. Reported success rates range from 68% to 93%, depending on case selection, treatment protocol, and evaluation criteria [1]. Long‐term success depends on adequate chemomechanical debridement followed by three‐dimensional sealing of the root canal space to prevent bacterial penetration [2]. Root canal sealers are intended to fill the space between the core material and dentinal walls, improving adaptation and reducing voids. Epoxy resin–based sealers are among the most widely used materials in contemporary endodontics [3].

Conventional obturation techniques typically combine gutta‐percha cones with a sealer placed using lateral or vertical compaction. These approaches rely on adequate canal shaping to create space for compaction and the placement of the core material. While shaping is essential for disinfection and obturation, it inevitably involves dentin removal, the extent of which depends on instrumentation strategy and canal morphology [4]. Furthermore, polymerization shrinkage and interfacial debonding of certain sealers may contribute to microgap formation at the sealer–dentin or sealer–gutta‐percha interface, potentially compromising the long‐term seal [4, 5]. To reduce the number of material interfaces and promote minimally invasive approaches, monobloc concepts and flowable obturation materials have been introduced [5, 6].

OdneFill (ODF) is a low‐viscosity, injectable, light‐curable hydrogel‐based root canal filling material designed to function as a monobloc obturation system. The material consists of photocrosslinkable methacrylate‐functionalized macromers that polymerize upon light activation, forming a cross‐linked hydrogel network. In contrast to conventional resin sealers that may undergo polymerization shrinkage, this hydrogel system exhibits volumetric stability with slight expansion during curing, potentially enhancing adaptation to canal walls. Its hydrophilic properties improve dentin wettability and interfacial contact [7], supporting adaptation in minimally prepared canals.

The mechanical and degradation behavior of hydrogel‐based materials differs fundamentally from that of rigid resin‐based sealers. Their structural integrity depends on cross‐link density and network stability rather than solely on polymer chain entanglement. Degradation of hydrogels primarily occurs through hydrolytic or enzymatic cleavage of susceptible bonds within the polymer backbone, such as ester linkages [8, 9]. Previous investigations have demonstrated that ODF maintains structural stability when exposed to bacterial enzymes and exhibits antimicrobial activity [10]. However, independent evaluation of its sealing performance and its resistance to structural degradation under simulated biological conditions remains limited.

Effective root canal sealing must be evaluated using methods that reflect clinically relevant failure mechanisms. Various microleakage models have been described, including dye penetration, fluid filtration, glucose diffusion, and bacterial leakage systems [11–14]. Dye penetration methods are frequently used because of their simplicity; however, they may overestimate leakage in hydrophilic materials due to molecular diffusion through polymer networks that are impermeable to bacteria [14]. In contrast, bacterial leakage models assess the ability of obturation materials to prevent penetration of viable microorganisms, thereby more closely simulating reinfection processes observed in clinical failures [14–17].

The bacterial leakage model has been widely applied in endodontic research and is considered more biologically meaningful than dye‐based methods when evaluating resistance to microbial penetration [13]. Because persistent or secondary infection with microorganisms such as *Enterococcus faecalis* is strongly associated with endodontic treatment failure [15–17], a two‐chamber bacterial penetration system provides a relevant experimental framework for assessing sealing integrity.

In addition to sealing performance, long‐term clinical success requires structural stability of the obturation material. Root canal filling materials are exposed to hydrolytic, enzymatic, oxidative, and microbial challenges within the canal environment [18, 19]. Methacrylate resin–based sealers have been reported to undergo hydrolytic degradation over time, which may compromise structural integrity and contribute to leakage or inflammatory reactions [20]. In hydrogel systems, degradation is often reflected by enlargement of pore size resulting from cross‐link cleavage and network loosening [8, 9, 21]. Quantitative pore size analysis using Feret diameter measurements enables sensitive detection of early microstructural changes that may precede macroscopic material deterioration.

Therefore, the present study was aimed at evaluating both the sealing ability and degradation resistance of a hydrogel‐based obturation material under controlled in vitro conditions. First, the time to bacterial penetration of teeth obturated with ODF was compared with that of teeth obturated with gutta‐percha combined with an epoxy resin–based sealer using *E. faecalis* as the test organism [22]. Second, the effects of different incubation media on hydrogel degradation were assessed using scanning electron microscopy (SEM) and quantitative pore size analysis. The null hypotheses were as follows: (I) no significant difference in bacterial leakage between the hydrogel‐based material and the conventional gutta‐percha/sealer system, and (II) incubation medium and time do not significantly influence hydrogel pore structure.

## 2. Materials and Methods

### 2.1. Specimen Preparation

A total of 40 extracted, single‐rooted bovine incisors with a standardized working length (WL) of 18 mm were used in this study. According to the manufacturer′s instructions, the teeth were prepared up to ISO #30/04 using iRace files (FKG Dentaire SA, La Chaux‐de‐Fonds, Switzerland). They were irrigated three times with 2 mL of 1% sodium hypochlorite (NaOCl) between each file, totaling 6 mL of NaOCl for each specimen. The canals were subsequently rinsed with 2.5 mL of a 17% EDTA solution for 1 min to remove the smear layer, followed by another rinse with 2 mL of 1% NaOCl (three times, totaling 6 mL). To avoid interaction between the hypochlorite and the test organisms, the canals underwent a final rinse with 2 mL of distilled water three times, each for 1 min (total volume: 6 mL). Three specimens were lost due to anatomic complications encountered during preparation and were discarded from the study. Teeth were sterilized using an ethylene oxide gas sterilizer (Steri‐Vac 4XL, 3 M, United States) at 37°C for 5.5 h and stored in PBS. All subsequent procedures were conducted under aseptic conditions in a laminar‐flow environment. After preparation and sterilization, specimens were dried using paper points (Roeko, Coltene/Whaledent GmbH + Co. KG, Langenau, Germany) and randomly allocated to the experimental groups using a computer‐generated randomization sequence. Allocation was performed by an investigator not involved in leakage assessment. Group sizes were determined based on the primary objective of comparing the sealing performance of the hydrogel‐based material with that of the conventional gutta‐percha plus epoxy resin sealer group. A larger positive control group was included to validate the leakage model, whereas the negative control group was limited to sterility verification. An a priori sample size calculation was performed using mean ± standard deviation leakage time data derived from a previously published two‐chamber *E. faecalis* bacterial leakage model [23]. The reported intergroup differences corresponded to large effect sizes (Cohen′s *d* ≥ 1.0). Assuming a two‐sided *α* level of 0.05 and 80% statistical power, a minimum of eight specimens per group was required. To account for potential specimen loss, biological variability of extracted teeth, and the longitudinal nature of leakage experiments, group sizes were increased to 10–12 specimens, thereby ensuring sufficient power and robust survival estimates for detecting clinically meaningful differences in leakage resistance.

The teeth were categorized into four distinct groups, as detailed in Table 1. The first three groups were incubated in a solution of *E. faecalis*. After chemomechanical preparation up to size 30/0.04 taper using stainless‐steel K‐files (Kerr Endodontics, Orange, CA, United States) and NiTi rotary files (HyFlex CM/EDM, Coltene/Whaledent AG, Altstätten, Switzerland), the canals were dried with sterile paper points prior to obturation. This included 12 teeth obturated with gutta‐percha cones (Coltene/Whaledent GmbH + Co. KG, Langenau, Germany) and AH Plus (Dentsply DeTrey GmbH, Konstanz, Germany), utilizing the cold lateral compaction technique as described previously [24]. AH Plus sealer (Dentsply DeTrey GmbH, Konstanz, Germany) was mixed in a 1:1 ratio on a glass slab using a metal spatula. A size 30, 0.04‐taper gutta‐percha master cone (Coltene/Whaledent GmbH + Co. KG, Langenau, Germany) was selected to approximate the shape of the prepared canal and fitted to the WL with confirmed tug‐back. If the cone bound short of the WL, a smaller cone was selected; if it reached WL without adequate tug‐back, 1 mm was trimmed from the tip before reassessment. The master cone was lightly coated with AH Plus sealer and placed into the canal. Lateral compaction was performed using a size C finger spreader (Dentsply Maillefer, Ballaigues, Switzerland). Size 30 accessory gutta‐percha cones (Coltene/Whaledent GmbH + Co. KG, Langenau, Germany) were sequentially inserted and condensed using a size B spreader (Dentsply Maillefer, Ballaigues, Switzerland) until no further cones could be accommodated. A Glick #1 plugger (Hu‐Friedy, Chicago, IL, United States) was heated and used to remove excess coronal gutta‐percha.

**Table 1:** Sample size, median survival time, and mean survival time ± standard error (SE) for groups exposed to *E. faecalis* or sterile BHI medium.

Group #	Group name	Obturation type	Incubation medium	*n*	Median (days)	*M* *e* *a* *n* ± *S* *E*(days)
1	Gutta‐percha	Gutta‐percha + AH Plus	*E. faecalis*	12	22	38.39 ± 8.37^a^
2	Odne®Fill	Odne®Fill	*E. faecalis*	10	121	84.71 ± 4.76^b^
3	Positive control	Empty canal	*E. faecalis*	11	1	0.71 ± 2.55^c^
4	Negative control	Odne®Fill	Sterile BHI	4	139	99.82 ± 1.06^d^

*Note:* Different superscripts (a–d) indicate statistically significant differences between groups.

The second group comprised 10 teeth obturated with ODF (Odne AG, Dübendorf, Switzerland) (Group 2), which was injected into the dried canal space according to the manufacturer′s instructions and polymerized using a dental light‐curing unit to ensure complete curing of the material. Eleven teeth were left empty and acted as the positive control (Group 3). The negative control (Group 4) included four teeth obturated with ODF and incubated in a sterile brain heart infusion (BHI) medium.

The bovine teeth used in this study were collected from animals slaughtered for commercial food production at a local abattoir (SBZ Schlachtbetrieb Zürich AG, Zurich, Switzerland). No animals were sacrificed specifically for research purposes. According to Swiss regulations, the use of such biological material does not fall under the Human Research Act. Therefore, formal approval by the Cantonal Ethics Committee was not required (BASEC‐Nr. Req‐2020‐00786).

### 2.2. Bacterial Leakage

**Figure 1:**
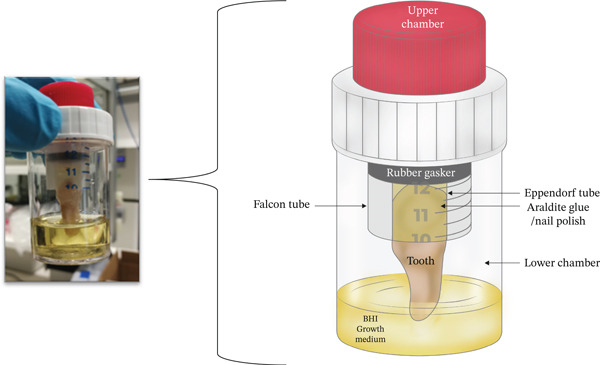
Photo and schematic showing the experimental microleakage setup.

Two layers of regular nail polish were applied to the outer surfaces of the teeth, ensuring that the apical 2–3 mm and the coronal access of the root canal remained untouched. The teeth were then positioned inside sectioned Eppendorf tubes, and the gaps between the teeth and the tube walls were sealed with two applications of a 2‐component glue (Araldite Rapid, Human Advanced Materials, Basel, Switzerland). A rubber ring fitted around the Eppendorf tube created a secure seal upon its insertion into a Falcon tube. Subsequently, the Falcon tube was placed within a larger chamber, as depicted in the accompanying diagram (Figure 1).

After obturation with the ODF material for both the negative control and the test samples, 500 *μ*L of PBS was added to the upper chamber of each sample to maintain constant hydration. All setups were incubated at 37°C for 24 h to approximate physiological conditions. Sterile BHI medium (5–7 mL) was added to the lower chambers, ensuring that the apex of each tooth was in contact with the solution. The PBS solution was subsequently removed from all samples. For five tooth setups obturated with ODF (serving as the negative control), 500 *μ*L of sterile BHI medium was introduced into the upper chamber. For the remaining 10 teeth, 500 *μ*L of *E. faecalis* solution, pregrown in BHI medium for 24 h at 37°C (with an optical density of 0.1 at *λ* = 600 nm in sterile BHI medium), was added to the upper chamber. The other samples, consisting of 10 unfilled teeth and 10 filled with gutta‐percha, were assessed alongside 500 *μ*L of *E. faecalis* solution. The samples were then incubated at 37°C. Every 2 days, 300 *μ*L of solution from the upper chamber was removed and replaced with 300 *μ*L of sterile BHI medium. The turbidity of the medium in the lower chamber was visually assessed daily by two independent observers under standardized lighting conditions, and the presence of visible cloudiness compared with sterile controls was recorded as a positive leakage event. Both observers were blinded to group allocation during turbidity assessment. The time to observe turbidity served as an indicator of bacterial leakage into the lower chamber. Turbidity assessment is a qualitative measure of viable bacterial penetration and is widely used in two‐chamber leakage models to determine time‐to‐event outcomes rather than quantitative bacterial load [11, 13, 14].

### 2.3. Degradation and Pore Size Analysis

Thirty samples of photocured ODF were prepared by adding 40 *μ*L of obturation material to the cap of a 200 *μ*L microcentrifuge tube and polymerizing for 1 min with a dental light‐curing device (FUSION 5, DentLight Inc., Plano, TX, United States). The samples were placed into 48‐well plates and soaked in 1 mL of PBS (AppliChem, ITW Reagents, S.R.L., Milano, Italy) each for 24 h at 37°C and divided into five groups (six samples each). The five groups were then incubated in 1 mL of the following solutions at 37°C: PBS (AppliChem, negative control), BHI broth (Sigma‐Aldrich, Merck & Cie, Buchs, Switzerland), *E. faecalis* suspension (OD600 = 0.1) in BHI broth, sterile‐filtered human saliva (0.22 *μ*m filter, 1:2 diluted with PBS), and 100 mM sodium carbonate buffer at pH 10 (positive control). For the *E. faecalis* group, every other day, 800 *μ*L of the bacterial solution was discarded and replaced by fresh, sterile BHI broth, and filtered saliva was replenished from stock solutions.

Two specimens were extracted from each group after 1, 8, and 30 days of incubation in the solutions. The samples were washed twice with 1 mL of PBS and rinsed in a 1 mL PBS‐filled ultrasonic bath for 10 s. The samples were then removed from the PBS solution, visually inspected for signs of degradation or surface alterations, and stored in 1 mL of PBS. For SEM analysis, the samples were dehydrated using an ascending series of ethanol concentrations. After the ethanol was replaced with liquid CO_2_, the samples underwent critical point drying with CO_2_, were mounted for SEM, and then were coated with 4 nm of a platinum/palladium (80/20) mixture. The drying resulted in ~30% shrinkage of the hydrogels as measured through linear shrinkage. Surface images were captured in secondary electron mode at 4 kV. Overview images were taken, followed by higher magnification (×10,000) images of five randomly selected surface regions. Images that showcased the hydrogel network were subsequently chosen for further analysis.

In order to quantify surface erosion, regions of the images that highlighted the hydrogel network were cropped and binarized using a manually determined threshold to differentiate between holes and network structures. The “Analyze Particles” function in Fiji (ImageJ) was then used with a minimum size setting of 10 pixels [12]. Based on SEM calibration at ×10,000 magnification, one pixel corresponded to 0.0168 *μ*m, resulting in a minimum detectable particle area of approximately 0.028 *μ*m^2^. The Feret diameter of each particle was recorded, enabling quantification of degradation via pore size measurements [25, 26]. An increase in average pore size would indicate hydrolysis or enzymatic cleavage of the cross‐links in the hydrogel [9, 10, 21]. Using Fiji (ImageJ), particles larger than 10 pixels were identified, and their Feret diameter was measured across all samples and time points.

### 2.4. Statistical Analysis

Statistical analyses were performed using SPSS software (IBM SPSS Statistics for Windows, v22; IBM Corp). Normality of the data distributions was assessed using *Q*–*Q* plots and Shapiro–Wilk tests. Homogeneity of variances was evaluated using Levene′s test. As the assumptions of normality and equal variance were not met, nonparametric statistical methods were used for inferential analyses.

For the bacterial leakage experiment, survival times were analyzed using Kaplan–Meier survival analysis, and differences between groups were assessed using nonparametric comparison tests. Pairwise comparisons were performed where appropriate. Survival outcomes are presented as median survival time and mean ± standard error (SE).

For the degradation and pore size analysis, time‐dependent differences within each incubation medium were evaluated using the Kruskal–Wallis test. When significant differences were detected, post hoc pairwise comparisons were conducted using the Mann–Whitney *U* test with Holm′s adjustment for multiple comparisons. Effect sizes (*r*) were calculated for pairwise comparisons to quantify the magnitude of differences. The significance level was set at *α* = 0.05.

## 3. Results

### 3.1. Distributional Assessment (*Q*–*Q*/*P*–*P* Plots)

Visual inspection of the *Q*–*Q* plots revealed systematic departures from the theoretical normal line, particularly in the distribution tails, indicating non‐normal behavior across the evaluated outcomes. The corresponding *P*–*P* plots also showed deviations in cumulative probabilities from the expected diagonal reference, supporting the graphical evidence of non‐normality. These graphical observations are consistent with the decision to use nonparametric tests for group comparisons and time‐dependent analyses (as outlined in the Statistical Analysis section).

### 3.2. Bacterial Leakage

**Figure 2:**
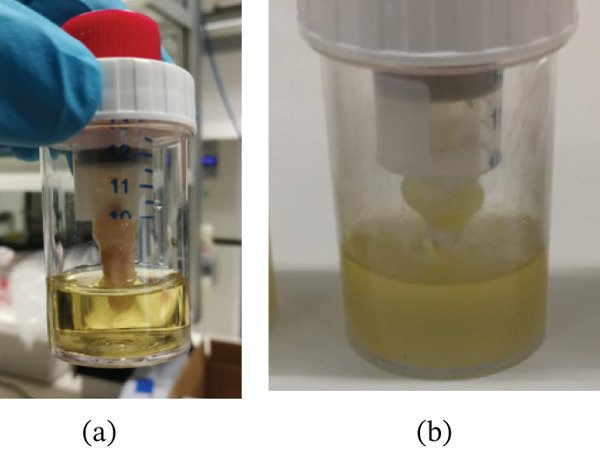
Representative images of turbidity used to identify leakage. (a) Negative control showing no turbidity throughout incubation, confirming system sterility and chamber integrity. (b) Positive control (unfilled canal) after 1 day of incubation. Clear turbidity is visible in the lower chamber, demonstrating leakage. This turbidity served as the criterion for determining leakage events.

**Figure 3:**
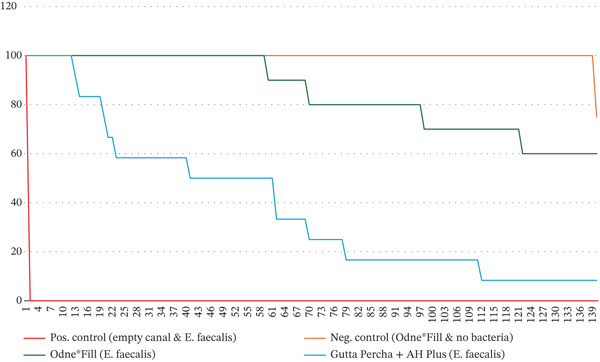
Survival curves of Odne®Fill and gutta‐percha + AH Plus. The leakage rate is faster in teeth filled with gutta‐percha and AH Plus compared to Odne®Fill.

Representative images demonstrating turbidity in positive controls and the absence of turbidity in negative controls are shown in Figure 2. These images illustrate the visual criteria used to identify the onset of leakage in the lower chamber. Figure 3 presents the survival curves under different conditions after incubation for up to 139 days at 37°C. The experimental setup was compatible with the use of ODF in this in vitro bacterial leakage model. The negative control exhibited leakage at 139 days, which occurred later than the initial failures observed in both the gutta‐percha + AH Plus and ODF groups. After 1 day of incubation, the positive control involving an unfilled root canal showed turbidity in the lower chamber.

In Figure 3, the survival curve for gutta‐percha + AH Plus declines earlier and more steeply than the ODF curve, indicating earlier and more frequent leakage events in the conventional obturation group, whereas ODF demonstrates a delayed decline and prolonged leakage‐free survival.

The first samples failed after 11 days for the gutta‐percha + AH Plus and after 58 days for the ODF. Additionally, 40% of the gutta‐percha samples failed after 22 days, while 40% of the ODF samples failed after 121 days of incubation. Thus, ODF delayed both the first observed leakage event (58 vs. 11 days) and the time to 40% failure (121 vs. 22 days) compared with gutta‐percha + AH Plus.

The median survival time was 22 days for GP + AH Plus and 121 days for ODF. The corresponding mean survival times were 38.39 ± 8.37 days and 84.71 ± 4.76 days, respectively. These results are summarized in Table 1.

Compared with the gutta‐percha + AH Plus group (mean 38.39 ± 28.99 days; median 22 days), the ODF group demonstrated a higher mean survival time (84.71 ± 15.05 days) and a substantially longer median survival time (121 days). The larger standard deviation in the gutta‐percha + AH Plus group reflects greater dispersion in individual leakage times compared with ODF. The discrepancy between the mean and median values in the gutta‐percha group suggests a right‐skewed distribution, with early clustering of failures and fewer later events contributing to the broader variability observed in this group.

Following the failure of the negative control, the experiment was terminated. Nonparametric tests indicated significant differences between the groups (*p* < 0.001). All pairwise comparisons between groups were statistically significant (all *p* < 0.001), confirming differences in leakage‐free survival among all experimental and control groups (Figure 3).

Overall, both the numerical outcomes (first failure times, 40% failure times, and mean/median survival) and the survival curve separation in Figure 3 consistently demonstrate prolonged leakage resistance for ODF relative to gutta‐percha + AH Plus in this model.

### 3.3. Degradation and Pore Size Analysis

**Figure 4:**
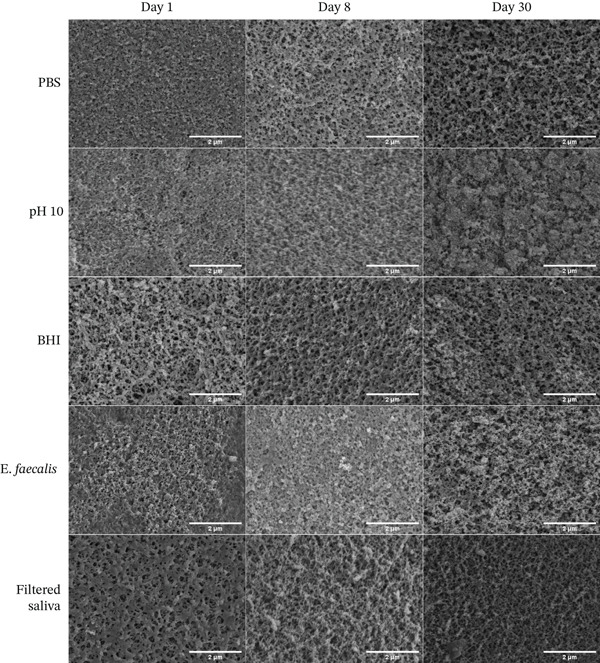
Surfaces of hydrogel samples after 1‐, 8‐, and 30‐day incubation in PBS (negative control), carbonate buffer at pH 10 (positive control), BHI, *E. faecalis* in BHI, or filtered saliva. At pH 10 (positive control), significant disintegration of the network was observed. For all other conditions, the hydrogel network remained intact, resembling the behavior of the negative control.

After removal from the solutions, all samples appeared mechanically intact and showed no visible changes in overall size, hardness, or surface structure. SEM analysis enabled a detailed examination of surface morphology. A cross‐linked network was observed for specimens incubated in PBS on Day 1 and remained visually intact over 30 days of incubation. Samples incubated in a pH 10 buffer for 30 days displayed a more disordered surface structure. Specimens incubated in BHI, filtered saliva, and *E. faecalis* suspension showed no visually detectable structural disruption compared to PBS (Table 2 and Figure 4).

**Table 2:** Time‐dependent changes in Feret diameter (micrometers) of Odne®Fill after incubation in different media. Mean ± standard deviation (SD) values are presented for Day 1, Day 8, and Day 30. Time‐dependent differences within each incubation medium were analyzed using the Kruskal–Wallis test, followed by Holm‐adjusted Mann–Whitney *U* pairwise comparisons. The effect size (*r*) range reflects the minimum and maximum *r* values observed among significant pairwise comparisons within each medium.

Incubation medium	Day 1 (*m* *e* *a* *n* ± *S* *D*)	Day 8 (*m* *e* *a* *n* ± *S* *D*)	Day 30 (*m* *e* *a* *n* ± *S* *D*)	Kruskal–Wallis *H* (*p* value)	Significant pairwise comparisons (Holm‐adjusted)	Effect size range (*r*)	*Δ* Day 1–30 (*μ*m)
PBS	0.122 ± 0.031	0.151 ± 0.055	0.134 ± 0.042	54.47 (*p* < 0.001)	All time points differed (*p* < 0.001)	0.127–0.273	+0.012
BHI	0.148 ± 0.054	0.133 ± 0.043	0.171 ± 0.079	60.00 (*p* < 0.001)	All time points differed (*p* < 0.001)	0.125–0.249	+0.023
*E. faecalis*	0.128 ± 0.039	0.141 ± 0.050	0.166 ± 0.075	68.77 (*p* < 0.001)	All time points differed (*p* < 0.001)	0.134–0.280	+0.038
Filtered saliva	0.133 ± 0.039	0.151 ± 0.061	0.154 ± 0.060	31.67 (*p* < 0.001)	Day 1 vs. 8 (*p* < 0.001); Day 1 vs. 30 (*p* < 0.001); Day 8 vs. 30 (*p* = 0.346)	0.030–0.172	+0.021
pH 10 buffer	0.132 ± 0.047	0.169 ± 0.076	0.232 ± 0.185	69.30 (*p* < 0.001)	All time points differed (*p* < 0.001)	0.119–0.323	+0.100

*Note: Δ* Day 1–30 represents the absolute change in mean Feret diameter between Day 1 and Day 30.

Accordingly, Figure 4 visually indicates preserved network morphology in PBS, BHI, *E. faecalis*, and filtered saliva across time points, whereas the pH 10 condition shows comparatively greater surface irregularity by Day 30.

**Figure 5:**
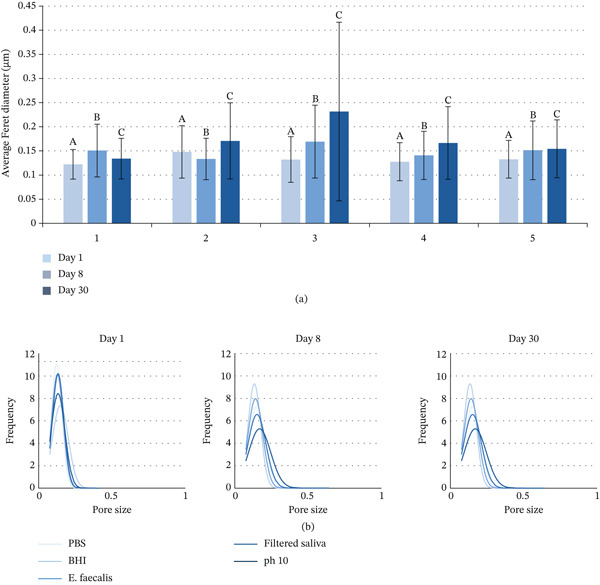
(a) Average Feret diameter (micrometers) representing pore size of Odne®Fill samples after 1, 8, and 30 days of incubation in PBS, BHI, *E. faecalis* suspension, filtered saliva, and pH 10 buffer. Bars represent mean ± standard deviation. Different uppercase letters (A–C) above the error bars indicate statistically significant differences between time points within the same incubation medium (Holm‐adjusted Mann–Whitney *U* test, *p* < 0.05). Identical letters indicate no statistically significant difference. (b) Probability density functions showing pore size distribution of Odne®Fill samples after 1, 8, and 30 days of incubation in the respective media.

Figure 5a illustrates the average pore size (Feret diameter, micrometers) over time for each incubation medium, and Figure 5b presents the corresponding pore size distributions as probability density functions. The mean ± SD Feret diameters (micrometers) at Day 1, Day 8, and Day 30 were 0.122 ± 0.031, 0.151 ± 0.055, and 0.134 ± 0.042 for PBS; 0.148 ± 0.054, 0.133 ± 0.043, and 0.171 ± 0.079 for BHI; 0.128 ± 0.039, 0.141 ± 0.050, and 0.166 ± 0.075 for *E. faecalis*; 0.133 ± 0.039, 0.151 ± 0.061, and 0.154 ± 0.060 for filtered saliva; and 0.132 ± 0.047, 0.169 ± 0.076, and 0.232 ± 0.185 for pH 10 buffer, respectively.

Table 2 and Figure 5a provide a structured overview of these mean ± SD values across time points and media. Across PBS, BHI, *E. faecalis*, and filtered saliva, mean pore sizes showed only modest fluctuations over time, whereas the pH 10 buffer group demonstrated the largest increase in mean pore size by Day 30 and the greatest dispersion (SD 0.185 at Day 30), indicating increased heterogeneity under alkaline stress.

Figure 5b supports this interpretation: Pore size distributions for PBS, BHI, *E. faecalis*, and filtered saliva show substantial overlap across time points, while the pH 10 condition shows a clear rightward shift and distribution broadening at Day 30, consistent with enlargement and increased variability of pore sizes.

Kruskal–Wallis tests revealed significant time‐dependent differences within each incubation medium (PBS: *H* = 54.47, *p* < 0.001; BHI: *H* = 60.00, *p* < 0.001; *E. faecalis*: *H* = 68.77, *p* < 0.001; filtered saliva: *H* = 31.67, *p* < 0.001; pH 10 buffer: *H* = 69.30, *p* < 0.001). Holm‐adjusted pairwise Mann–Whitney *U* tests confirmed significant differences between most time points. For the pH 10 buffer, all pairwise comparisons were significant (Day 1 vs. Day 8: *p* < 0.001, *r* = 0.245; Day 1 vs. Day 30: *p* < 0.001, *r* = 0.323; Day 8 vs. Day 30: *p* < 0.001, *r* = 0.119). In contrast, for filtered saliva, the comparison between Day 8 and Day 30 was not significant (*p* = 0.346, *r* = 0.030), while all other comparisons in PBS, BHI, and *E. faecalis* reached statistical significance (all *p* < 0.001; *r* values ranging from 0.125 to 0.280) (Table 2).

Thus, statistical testing identified time effects within each medium, while effect sizes and descriptive changes (Table 2) contextualize the magnitude of these differences. Notably, filtered saliva showed no detectable difference between Day 8 and Day 30 (*p* = 0.346, *r* = 0.030), consistent with the minimal separation of mean values and overlapping distributions in Figure 5.

The largest absolute increase in mean pore size over time was observed in the pH 10 buffer group (*Δ* = +0.100 * μ*m from Day 1 to Day 30), whereas changes in the other media remained below 0.05 *μ*m. The corresponding pore size distributions are shown in Figure 5b.

This indicates that the most pronounced pore enlargement occurred under alkaline (pH 10) conditions, while pore size changes under PBS, BHI, *E. faecalis*, and filtered saliva remained limited in absolute terms over the 30‐day period.

Although statistically significant time effects were detected across incubation media, the magnitude of pore enlargement under biologically relevant conditions (PBS, BHI, *E. faecalis*, and filtered saliva) remained limited compared with the pronounced increase observed under alkaline conditions.

Taken together, Figures 4 and 5 and Table 2 show that ODF specimens largely preserved network morphology and exhibited only minor pore size changes under biologically relevant media, whereas alkaline pH 10 exposure produced the clearest structural signal—an increase in mean pore size and a broader pore size distribution by Day 30.

## 4. Discussion

The present study was aimed at evaluating the functional sealing performance and structural stability of a light‐curable hydrogel‐based obturation material (ODF) by combining a time‐to‐event bacterial leakage model with quantitative SEM pore analysis. This integrated design enabled simultaneous assessment of resistance to microbial penetration and microstructural stability under controlled in vitro conditions.

The principal findings demonstrate that ODF exhibited prolonged leakage‐free survival compared with gutta‐percha combined with an epoxy resin–based sealer. In addition, hydrogel pore architecture showed time‐dependent changes that varied according to incubation environment, with more pronounced structural modification under alkaline exposure and comparatively limited alteration in biologically relevant media.

Based on these findings, the first null hypothesis, stating that no difference in bacterial leakage would exist between ODF and the conventional gutta‐percha/sealer system, is rejected. The separation of survival distributions indicates that obturation material selection influenced resistance to bacterial penetration under the tested conditions. The second null hypothesis, stating that incubation medium and time would not influence hydrogel pore structure, is partially rejected. Although structural changes were detected, their magnitude and biological relevance were strongly environment‐dependent and were primarily evident under nonphysiological alkaline stress rather than under simulated biological exposure [8, 9, 21].

Persistent microbial infection is a principal cause of endodontic treatment failure [15–17], and *E. faecalis* is frequently associated with persistent periradicular disease and retreatment cases [16, 17, 22]. Resistance to bacterial penetration, therefore, represents a clinically meaningful outcome parameter. The improved performance of ODF may be attributed to the formation of a continuous hydrogel network that can intimately adapt to the canal walls, consistent with the monoblock concept described in the endodontic literature [5]. Interfacial integrity and reduced gap formation are recognized determinants of leakage behavior [4, 5, 20].

Bacterial leakage models provide a biologically relevant alternative to dye penetration tests, particularly for hydrophilic materials, where low‐molecular‐weight tracers may diffuse through polymer networks and exaggerate leakage [11–14]. Kaplan–Meier–based survival analysis has been widely applied in comparative leakage investigations and allows appropriate modeling of time‐to‐event outcomes [27].

The conventional comparator was obturated using cold lateral compaction with gutta‐percha and an epoxy resin–based sealer. This technique remains widely used in laboratory investigations and enables reproducible material comparison [5, 24]. However, the obturation technique influences adaptation, void formation, and sealer distribution [4, 5]. Contemporary evidence indicates that warm vertical compaction or thermoplasticized techniques may enhance gutta‐percha adaptation in certain canal morphologies [28, 29]. In this context, previous bacterial leakage investigations have reported variable performance of gutta‐percha/AH Plus depending on experimental design. Oliveira et al. demonstrated that although AH Plus provided acceptable sealing ability, bacterial penetration still occurred over time, and differences among sealers were influenced by the specific leakage model and observation period [30]. Conversely, Monticelli et al. reported that obturation technique significantly affected bacterial leakage outcomes, with certain warm vertical approaches showing reduced leakage compared with single‐cone or lateral compaction methods [31]. These findings suggest that the leakage performance of GP/AH Plus is technique‐sensitive and model‐dependent. Accordingly, the present findings should be interpreted as a controlled comparison under standardized lateral compaction conditions rather than as a universal hierarchy of obturation strategies.

The two‐chamber bacterial leakage system, although extensively used in endodontic materials research [11–14, 27], represents a simplified experimental model. The monospecies challenge does not reproduce the polymicrobial biofilm ecology characteristic of clinical endodontic infections [15–17, 22, 32]. Turbidity detection provides a qualitative endpoint reflecting time to detectable bacterial passage rather than quantitative bacterial burden [11, 13]. In addition, experimental variables such as apparatus sealing and inoculum standardization may influence absolute leakage timing. Leakage results should therefore be interpreted comparatively rather than as direct predictors of clinical failure.

Hydrogel degradation involves cleavage of cross‐links and enlargement of pore architecture [8, 21]. Interpretation of structural change requires consideration of both statistical detectability and biological magnitude [9, 18, 19]. The present findings indicate that environmental chemistry strongly influenced hydrogel microstructure, with more pronounced alteration under alkaline conditions and limited modification under biologically relevant media. These observations are consistent with prior evaluations of hydrogel stability in dental applications [10].

A methodological limitation concerns the limited number of independent hydrogel specimens analyzed per condition and time point in the SEM evaluation. Although multiple pore measurements were obtained from each specimen, these observations are nested within specimens and are therefore not fully independent biological replicates. Failure to account for such clustering may introduce pseudoreplication and overestimation of inferential precision [33]. High‐resolution surface imaging also does not fully characterize three‐dimensional bulk degradation behavior. Contemporary discussions in experimental design emphasize aligning statistical interpretation with data hierarchy and effect magnitude [34]. Accordingly, pore size findings should be interpreted as consistent structural trends rather than definitive population‐level estimates.

Group allocation in the leakage experiment was intentionally unbalanced to ensure adequate positive control validation and sterility verification, consistent with established leakage study designs [11, 13]. Unequal group sizes may influence variance structure and survival estimate precision. Although an a priori power analysis supported the primary comparison, the study was not designed to detect subtle secondary effects.

Taken together, the leakage and degradation findings indicate that ODF maintains structural integrity under simulated biological conditions and demonstrates extended resistance to bacterial penetration compared with a conventional gutta‐percha/sealer system within the limits of this in vitro design. However, the experimental conditions do not replicate mechanical loading, cyclic pH fluctuations, host immune responses, or complex multispecies biofilm dynamics present clinically [15–17]. Future investigations incorporating long‐term aging, acidic and enzymatic challenges, multispecies biofilm models, and mechanical fatigue simulation are required to determine whether the observed laboratory advantages translate into improved long‐term clinical outcomes [1].

## 5. Conclusions

This in vitro study demonstrates that the light‐curable hydrogel‐based obturation material provides prolonged resistance to *E. faecalis* penetration compared with gutta‐percha combined with an epoxy resin–based sealer in a bacterial leakage model. SEM analysis showed time‐dependent pore size changes, with meaningful enlargement primarily under alkaline conditions, while pore architecture remained comparatively stable in biologically relevant environments over 30 days. These findings indicate that the hydrogel maintained structural integrity and resistance to bacterial leakage under simulated conditions. Long‐term and in vivo studies are required to confirm clinical relevance.

## Author Contributions

Conceptualization: E.E., D.R., M.B., and A.S. Methodology: E.E., D.R., M.B., and A.S. Validation: E.E., D.R., I.B.P., M.B., A.S., T.F.E., and M.Ö. Formal analysis: E.E. and T.F.E. Investigation: D.R., I.B.P., M.B., and A.S. Data curation: E.E., D.R., I.B.P., M.B., and A.S. Writing—original draft preparation: E.E., T.F.E., and M.Ö. Writing—review and editing: T.F.E. and M.Ö.

## Funding

This project was partly supported by a grant from Innosuisse ‐ Swiss Innovation Agency, Zurich, Switzerland (Grant No. 45882.1 IP‐LS), where Lumendo Ltd (currently named Odne AG) was a project partner. Open access publishing facilitated by Universitat Zurich, as part of the Wiley ‐ Universitat Zurich agreement via the Consortium Of Swiss Academic Libraries.

## Disclosure

The funding body had no role in the study design, data collection and analysis, publication decision, or manuscript preparation. The potential conflicts of interest have been fully disclosed and do not affect the objectivity or integrity of the research findings.

## Ethics Statement

The authors have nothing to report.

## Conflicts of Interest

The authors, Else Ellermann, Daniel Richter, Ignasi Belda Punzano, Andreas Schmocker, and Mark Bispinghoff, are now employees of Odne AG, the manufacturer of the hydrogel‐based obturation material investigated in this study (OdneFill). In addition, some authors are inventors of patents related to this product.

## Data Availability

The data is available upon request.
